# CYP3A-Mediated Metabolism of Zastaprazan in Humans and Associated Drug–Drug Interactions

**DOI:** 10.3390/pharmaceutics18060718

**Published:** 2026-06-10

**Authors:** Kai-Juan Cao, Long Fu, Yu-Chen Sun, Jian Meng, Qin Huang, De-Cheng Deng, Hai-Tang Hu, Zhi-Hui Han, Gang Guo, Xue Zhou, Xiao-Yan Chen

**Affiliations:** 1 State Key Laboratory of Drug Research, Shanghai Institute of Materia Medica, Chinese Academy of Sciences, Shanghai 201203, China; caokaijuan@simm.ac.cn (K.-J.C.); sunyuchen@simm.ac.cn (Y.-C.S.); jmeng@simm.ac.cn (J.M.); guogang@simm.ac.cn (G.G.); 2 University of Chinese Academy of Sciences, Beijing 100049, China; 3 Livzon Pharmaceutical Group Inc., Zhuhai 519000, China; fulong@livzon.cn (L.F.); huangqin01@livzon.cn (Q.H.); dengdecheng@livzon.cn (D.-C.D.); huhaitang@livzon.cn (H.-T.H.); hanzhihui@livzon.cn (Z.-H.H.); 4 School of Pharmacy, Fudan University, Shanghai 200433, China

**Keywords:** [^14^C]JP-1366, CYP3A-mediated metabolism, physiologically based pharmacokinetics, drug–drug interaction, gastroesophageal reflux disease

## Abstract

**Background/Objectives**: Zastaprazan (JP-1366) is a novel potassium-competitive acid blocker (P-CAB) used for the treatment of gastroesophageal reflux disease (GERD). To date, its metabolic pathways and metabolism-related drug–drug interactions (DDIs) in humans remain incompletely elucidated. This study aimed to determine the relative contributions (f_m_) of cytochrome P450 isoforms to JP-1366 elimination and assess its DDI potential. **Methods**/**Results**: In vitro metabolic studies using human liver microsomes (HLMs) revealed that JP-1366 was first metabolized to M1, which subsequently underwent further oxidation, glucuronidation, and *N*-dealkylation. Mono-oxidation was estimated to contribute more than 46% to the overall metabolic clearance of JP-1366. Reaction phenotyping identified CYP3A as the major enzyme (f_m_ = 96.1%), followed by CYP1A2 (1.49%) and CYP2C9 (2.41%). By integrating in vitro data, clinical pharmacokinetic data and clarithromycin coadministration DDI data, a physiologically based pharmacokinetic (PBPK) model was developed and validated. Simulations predicted significant DDIs with strong CYP3A inhibitor (ketoconazole), with AUC ratios of 3.80. Moderate inhibitors (fluconazole and fluvoxamine) caused mild increases (AUC ratios: 1.14–1.74). Conversely, strong and moderate CYP3A inducers, rifampicin and efavirenz, produced pronounced DDIs, with AUC ratios of 0.22 and 0.50, respectively. Furthermore, simulations predicted that although JP-1366 functions as a CYP enzyme inhibitor, it would not cause clinically meaningful changes in the plasma exposure of corresponding CYP substrate drugs; however, potential interactions with CYP3A substrates still warranted consideration. **Conclusions**: JP-1366 is predominantly cleared via a CYP3A-dominated metabolic pathway. The PBPK simulations suggest that JP-1366 may be a moderately sensitive CYP3A substrate and a moderate inhibitor of sensitive CYP3A substrates, while its perpetrator DDI risk toward other major CYP pathways appears limited. These findings support caution or monitoring when JP-1366 is co-administered with strong CYP3A modulators or sensitive CYP3A substrates.

## 1. Introduction

Gastroesophageal reflux disease (GERD) is a common chronic gastrointestinal disorder caused by the reflux of gastric contents into the esophagus. It is characterized by typical symptoms such as heartburn and acid regurgitation, which can significantly impair patients’ quality of life [1]. Epidemiological studies have shown that the global prevalence of GERD is approximately 13.98% and a continuously increasing trend has been observed worldwide [2].

Proton pump inhibitors (PPIs) achieve potent acid suppression by irreversibly inhibiting the H^+^/K^+^-ATPase and have been recommended as the first-line therapy for GERD in multiple international clinical guidelines [3,4,5,6]. However, PPIs typically need 3–5 consecutive days to achieve maximal acid suppression [7]. Furthermore, most PPIs are extensively metabolized by hepatic cytochrome P450 2C19 (CYP2C19), an enzyme with marked genetic polymorphism [8]. Approximately 15–20% of Asian individuals are poor CYP2C19 metabolizers, resulting in omeprazole exposure up to fourfold higher than that in Caucasians and an increased risk of adverse effects. In addition, PPIs often lead to “nocturnal acid breakthrough” (NAB) [9].

Potassium-competitive acid blockers (P-CABs) inhibit gastric acid secretion by reversibly and competitively binding to the K^+^-binding site of the H^+^/K^+^-ATPase. As weak bases, P-CABs readily accumulate in gastric parietal cells, exhibiting faster, stronger and longer-acting acid suppression than PPIs, and effectively overcoming the problem of NAB [10]. To date, six P-CABs have been approved for marketing, including revaprazan, vonoprazan, tegoprazan, fexuprazan, keverprazan and zastaprazan [11,12,13,14,15]. Zastaprazan (code: JP-1366) is currently seeking marketing approval in China. Pharmacological studies showed that JP-1366 exhibited similar inhibitory activity against the H^+^/K^+^-ATPase as vonoprazan (IC_50_ values: 21.6 nM vs. 19.5 nM), and greater potency than tegoprazan and fexuprazan (IC_50_ values: 150 nM and 79.4 nM, respectively) (in-house data). In Sprague-Dawley (SD) rats, JP-1366 inhibited basal gastric acid output by 91% compared with 66.9% for vonoprazan at an administration dose of 2 mg/kg [16].

In vitro studies demonstrated that JP-1366 was also a substrate of CYP3A, but not for the major uptake or efflux transporters, indicating that in vivo ADME properties of JP-1366 were mainly metabolism-driven [17]. Human metabolic studies further confirmed that JP-1366 was predominantly eliminated via metabolism in vivo. Oxidation and subsequent glucuronidation represented the major metabolic pathways in humans, and the mono-oxidative metabolite M1 was one of the major circulating metabolites [18]. Since P-CABs are commonly co-administered with clarithromycin (a strong CYP3A inhibitor) and amoxicillin for *Helicobacter pylori* eradication therapy in clinical practice, evaluation of potential CYP3A-mediated drug–drug interactions under clinically relevant combination conditions is important [19,20].

In the present study, to determine the fractional contributions (f_m_) of the CYP isoforms to total clearance, in vitro experiments were conducted using human liver microsomes (HLMs) supplemented with selective chemical inhibitors. Furthermore, a physiologically based pharmacokinetic (PBPK) model of JP-1366 was established and validated by integrating in vitro and clinical PK data to dynamically characterize its ADME processes in humans. The model was then applied to predict the DDI risk of JP-1366 as a victim drug (a substrate whose pharmacokinetic profile is modulated by another co-administered compound), as well as its potential effect on CYP3A substrates when acting as a perpetrator drug (the interacting agent that triggers these alterations via enzyme inhibition or induction). Collectively, these investigations provided a comprehensive understanding of the metabolism and dual DDI potential of JP-1366 as both a victim drug and a perpetrator drug, thereby offering a scientific foundation for its further development and safe clinical use.

## 2. Materials and Methods

### 2.1. Chemicals and Reagents

JP-1366 (content: 99.4%) and its hydroxylated metabolite M1 (content: 95.6%) were kindly provided by Livzon Pharmaceutical Group Inc. (Zhuhai, China). ^14^C]JP-1366 (radiochemical purity: 100.0%, specific radioactivity: 52.39 mCi/mmol) was synthesized by Wuxi Beta Pharmaceutical Technology Co., Ltd. (Wuxi, China).

Pooled HLMs were purchased from BIOIVT (Westbury, NY, USA). 1-Aminobenzotriazole (ABT), 6β-hydroxytestosterone and nicotinamide adenine dinucleotide phosphate (NADPH) were obtained from MCE (Monmouth Junction, NJ, USA). Uridine 5′-diphosphoglucuronic acid (UDPGA) was acquired from Sigma-Aldrich (Saint Louis, MO, USA). Alamethicin was provided by Shanghai Aladdin Biochemical Technology Co., Ltd. (Shanghai, China). α-Naphthoflavone (ANF), sulfaphenazole (SPZ), testosterone, phenacetin and diclofenac were purchased from Meilunbio (Dalian, China). Phosphate-buffered saline (PBS) was obtained from Corning (New York, NY, USA). 6β-hydroxytestosterone-d_6_ was purchased from ISOREAG Co., Ltd. (Shanghai, China). Acetaminophen was obtained from the National Institutes for Food and Drug Control (Beijing, China). Acetaminophen-d_4_ and 4′-hydroxymephenytoin-d_3_ were purchased from Toronto Research Chemicals (Toronto, ON, Canada). 4′-Hydroxydiclofenac was purchased from LGC Standards (Teddington, UK). The liquid scintillation cocktail was obtained from Shanghai Hongxiyuan Technology Co., Ltd. (Shanghai, China).

### 2.2. Study Design and Sample Collection

Pharmacokinetic datasets were obtained from an internal study report of a Phase I trial (NCT05194046) conducted in South Korea to evaluate DDI between JP-1366 and the strong CYP3A inhibitor clarithromycin [21]. These data were used strictly for secondary PBPK model development and verification; no new human subject interventions were conducted. The primary clinical study adhered to GCP guidelines and the Declaration of Helsinki, with informed consent secured from all participants before enrollment. Three dosing arms were designed for the study: T1, JP-1366 capsules (20 mg per capsule), 1 capsule twice daily for 5 consecutive days; T2, amoxicillin capsule (500 mg), 2 capsules + clarithromycin tablet (500 mg), 1 tablet, twice daily for 5 consecutive days; T3, JP-1366 capsule (20 mg), 1 capsule + amoxicillin capsule (500 mg), 2 capsule + clarithromycin tablet (500 mg), 1 tablet, twice daily for 5 consecutive days. Amoxicillin was co-administered to simulate the clinically relevant *Helicobacter pylori* eradication regimen containing clarithromycin. Since amoxicillin is not known to inhibit or induce CYP3A, it was not expected to meaningfully affect the metabolism of JP-1366. A total of 24 eligible subjects were enrolled.

### 2.3. In Vitro Metabolism of JP-1366 and M1 in HLMs

To investigate the correlation between in vitro and in vivo metabolic profiles of JP-1366, a reaction mixture containing [^14^C]JP-1366 (20 µM), HLMs (0.5 mg/mL), NADPH (1.0 mM) and ABT (1 mM) was prepared in 100 mM phosphate buffer (pH 7.4) supplemented with 3.2 mM MgCl_2_. Four incubation systems (100 μL each) were designed: (1) [^14^C]JP-1366 + HLMs + NADPH; (2) [^14^C]JP-1366 + HLMs; (3) [^14^C]JP-1366 + HLMs + NADPH + ABT; (4) PBS + [^14^C]JP-1366 as control. The reactions were initiated by the addition of [^14^C]JP-1366 after 3 min of pre-incubation and carried out for 60 min. After incubation, the samples were analyzed by an Acquity I-Class UPLC system (Waters, Milford, MA, USA) coupled with ARC™ on-line radioisotope detector (Model 3; AIM, Billerica, MA, USA). Metabolite identification was performed using ultra-performance liquid chromatography/quadrupole time-of-flight mass spectrometry (UPLC/Q-TOF MS).

To determine the in vitro metabolism of M1, a 100-μL incubation mixture containing HLMs (0.5 mg/mL), M1 (10 uM), and NADPH (1.0 mM) was prepared in 100 mM PBS (pH 7.4) supplemented with 3.2 mM MgCl_2_. The reaction protocol was identical to that used for [^14^C]JP-1366 incubation.

To identify the sequential glucuronidated metabolites, a 100-μL incubation mixture containing HLMs (0.5 mg/mL), M1 (10 μM), UDPGA (2.0 mM), and alamethicin (25 μg/mL) was prepared in 100 mM Tris-HCl buffer (pH 7.4) supplemented with 10 mM MgCl_2_. Prior to incubation, HLMs were mixed with alamethicin on ice for 15 min to permeabilize the membrane. The reactions were initiated by the addition of UDPGA after 3 min of pre-incubation and carried out for 1 h. Sample preparation followed the same procedures as described above. The supernatant was collected and analyzed by UPLC-Q/TOF MS.

### 2.4. Cytochrome P450 Reaction Phenotyping of JP-1366

Based on the previously reported JP-1366 data and our in vitro HLM incubation studies, CYP1A2, CYP2C9, and CYP3A4/5 were estimated to be the primary enzymes involved in the metabolism of JP-1366 [12,17,18]. To quantify the contributions of CYP1A2, CYP2C9, and CYP3A to the oxidative clearance of JP-1366, chemical inhibition experiments were conducted using the substrate depletion method, and the resulting data were processed via a coefficient matrix correction. Incubations were performed in 100 μL mixtures containing HLMs (0.025, 0.05, 0.1 and 0.2 mg/mL), JP-1366 (1 μM), and NADPH (1.0 mM) for 0, 5, 10, 20, 30, and 40 min.

Each reaction mixture containing the final concentration of 1.0 µM JP-1366, 0.1 mg/mL HLMs and either a specific inhibitor or no inhibitor was pre-incubated at 37 °C for 10 min. The specific inhibitors included ANF (2 µM, CYP1A2), SPZ (10 µM, CYP2C9), and AZA (3 µM, CYP3A). For the control group (no inhibitor), an equivalent volume of blank buffer was substituted for the inhibitor. Reactions were pre-incubated at 37 °C for 10 min and initiated by the addition of 1 mM NADPH. Aliquots were quenched with ice-cold acetonitrile at 0, 5, 10, 20, and 40 min. The remaining concentration of JP-1366 was determined using a validated LC-MS/MS method. All incubations were performed in duplicate.

To correct for potential off-target effects of the inhibitors, parallel incubations were conducted using established CYP probe substrates, following the same incubation protocol as that used for the reaction phenotyping of JP-1366. The probe substrates included phenacetin (50 µM for CYP1A2), diclofenac (10 µM for CYP2C9), and testosterone (100 µM for CYP3A). Reactions were terminated by adding ice-cold acetonitrile (100 µL) after 20 min (phenacetin, testosterone) or 10 min (diclofenac). The formation of the corresponding probe metabolites, acetaminophen for CYP1A2, 4′-hydroxydiclofenac for CYP2C9, and 6β-hydroxytestosterone for CYP3A, was quantified by LC-MS/MS, as shown in Supplementary Method S1.

### 2.5. LC-MS/MS Analysis for Quantification of JP-1366

A Triple Quad 5500 mass spectrometer (Sciex, Framingham, MA, USA) coupled with an LC-30AD HPLC system (Shimadzu, Kyoto, Japan) was used for quantification.

An Eclipse Plus C_18_ column (4.6 × 100 mm, 3.5 µm) was used for separation of JP-1366 and JP-1366-d_6_. Mobile phase A consisted of 5 mM ammonium acetate and 0.2% formic acid in water, and mobile phase B was acetonitrile. Gradient elution was performed at a flow rate of 0.8 mL/min as follows: 0–0.3 min, 40% B; 1–2 min, 95% B; 2.01 min, 40% B; 2.5 min, stop. The electrospray ionization source (ESI) was operated in positive ion mode with the nebulizer gas (GS1), heater gas (GS2), and curtain gas (CUR) set at 50, 50, and 40 psi, respectively. The ion spray potential was 5500 V and the source temperature was 500 °C. The quantitative transition ion pair of JP-1366 was *m*/*z* 363.2→257.1 with collision energy (CE) being 21 V, while JP-1366-d_6_ was *m*/*z* 369.2→125.2 with collision voltage (CE) being 80 V. The declustering potential (DP) was 60 V. The calibration curve of JP-1366 was linear over the concentration ranges of 1.00–500 ng/mL, with a lower limit of quantification of 1.00 ng/mL. Quality control samples (QC) were prepared at 3.00, 20.0, 250, and 400 ng/mL. The intra- and inter-batch precision and accuracy met the predefined acceptance criteria, with precision values not exceeding 14.3% and accuracy deviations within −14.7% to 13.5%. Extraction recovery, matrix effect, and stability were also evaluated and found to be acceptable.

### 2.6. Calculation of CYP Enzyme Contribution (f_m_) to Metabolism of JP-1366

The potential off-target effects of chemical inhibitors were evaluated using CYP marker reactions. The metabolite formation rates of CYP probe substrates were quantified in the absence and presence of each chemical inhibitor, denoted as *v_control,CYPa_* and *v_inhibiotrA__,CYPa_*, respectively. The fractional inhibition (*I*) of a given enzyme (*a*) by an inhibitor (*A*) was subsequently calculated using Equation (1):

(1) $$I_{inhibitorA,CYPa}=(v_{control,CYPa}-v_{inhibitorA,CYPa})/v_{control,CYPa}\times 100\%$$

where *v_control,CYPa_* represents the metabolite formation rate of the CYPa marker reaction in the HLM control incubation, and *v_inhibiotrA,CYPa_* represents the metabolite formation rate of the CYPa marker reaction in the presence of inhibitor A. These values were used to generate the inhibitor cross-reactivity matrix for subsequent correction of CYP fractional contributions.

The fractional metabolic contribution (f_m_) of individual CYP enzymes to the metabolism of JP-1366 was estimated using GraphPad Prism 9 software (GraphPad Software, San Diego, CA, USA). For the JP-1366 depletion assay, the natural logarithm of the remaining parent fraction ([ln(Ct/C0)]) was plotted against the incubation time, and linear regression analysis was performed. The depletion rate constant (k) was defined as the slope of the regression line. The uncorrected fractional contribution of each CYP enzyme was then calculated using Equation (2) [22]:

(2) $$f_{m,uncorrected}=[(k_{control}-k_{inhibitor})/k_{control}]\times 100\%$$

where *k_contro_*_l_ represents the depletion rate constant of JP-1366 in the HLM control incubation, and *k_inhibitor_* represents the depletion rate constant in the presence of each selective CYP inhibitor.

Equation (3) was employed to correct for the off-target effects of CYP inhibitors. Here, *I_inhibitorA__,CYPa_* represents the percentage inhibition of the target enzyme a by inhibitor A. *f_m,corrected,CYP__a_* represents the corrected fractional contribution of specific CYP isoform (e.g., CYPa to the metabolism of JP-1366) [22].

(3) $$\left[\begin{array}{ccc} I_{inhibitorA,CYPa} & I_{inhibitorA,CYPb} & I_{inhibitorA,CYPc}\\ I_{inhibitorB,CYPa} & I_{inhibitorB,CYPb} & I_{inhibitorB,CYPc}\\ I_{inhibitorC,CYPa} & I_{inhibitorC,CYPb} & I_{inhibitorC,CYPc} \end{array}\right]\times \left[\begin{array}{c} f_{m,corrected,CYPa}\\ f_{m,corrected,CYPb}\\ f_{m,corrected,CYPc} \end{array}\right]=\left[\begin{array}{c} f_{m,uncorrected,CYPa}\\ f_{m,uncorrected,CYPb}\\ f_{m,uncorrected,CYPc} \end{array}\right]$$

### 2.7. Prediction of Drug–Drug Interactions for JP-1366 Based on a PBPK Model

The JP-1366 PBPK model was developed in GastroPlus™ (version 9.8; Simulation Plus Inc., Lancaster, CA, USA), incorporating in vitro, in silico and in vivo data for JP-1366. The developed model was subsequently used to simulate potential DDIs, following oral coadministration of JP-1366 with CYP3A inhibitors and inducers or substrates. Detailed model parameters are listed in Table S1. The virtual subjects employed in the simulations were designed to mimic the study populations. Given that the software lacked a built-in virtual subject model for the Korean population that actually participated in the clinical trials, the closest Chinese virtual subject model was used as a surrogate and set based on the reported population characteristics.

### 2.8. Refinement and Validation of the PBPK Model of JP-1366 Pharmacokinetics

The built-in Advanced Compartmental Absorption and Transit (ACAT) module was utilized to describe the absorption process of orally administered JP-1366. The model integrates drug solubility, permeability, logP, particle size, dissociation constant (pKa), and other characteristics that describe dissolution, precipitation, and absorption during the drug’s passage through the gastrointestinal tract. JP-1366 was characterized by a pKa of 5.81, a LogP of 1.72, a reference solubility of 0.002 mg/mL at pH 6.8, and a plasma unbound fraction of 1.13%. The apparent permeability (P_app_) across the Caco-2 cells was 2.3 × 10^−5^ cm/s, which was used to predict the effective intestinal permeability of 3.55 × 10^−4^ cm/s (Absca method) in humans. The solubility data at different pH values were used to simulate the dissolution behavior of JP-1366. Distribution was described using a whole-body PBPK model, with tissue-to-plasma partition coefficients calculated by the Poulin–Theil homogeneous method, and the blood-to-plasma ratio was set to 0.55. For the elimination module, both metabolism and renal excretion were considered. Hepatic metabolism was incorporated as the major clearance pathway, with CYP3A assigned as the predominant enzyme based on in-house HLM metabolic stability and reaction phenotyping data (in-house data). The CYP3A K_m_ and V_max_ values were 13.7 μM and 7.91 nmol/min/mg protein, respectively. CYP2C9 and CYP1A2 were included as minor metabolic pathways. Renal clearance was set to 0.002 L/h based on clinical PK data. Detailed model parameters are listed in Table S1.

The model was verified by simulating data from additional studies (not used during model development) across different dose levels and administration regimens. To assess the accuracy of the JP-1366 PBPK model, both single-dose (5 mg, 10 mg, 10 mg-fed, 20 mg, 40 mg and 60 mg) and multiple-dose (5 mg, 10 mg, 20 mg and 40 mg) regimens were tested. Validation of the established JP-1366 model was based on clinical data obtained from published studies. Detailed clinical study data used in PBPK model development and verification are shown in Table S2. The accuracy of the PBPK model was verified by comparing the predicted and observed PK parameters, with an acceptance criterion of a fold error within 0.5–2.

### 2.9. Validation of the PBPK Model of JP-1366 as a Victim with Clarithromycin

The fraction of JP-1366 metabolized by CYP3A (f_m,CYP3A_) was verified using a clinical DDI study with clarithromycin, a strong CYP3A inhibitor. The doses and administration regimens of the drugs were selected to align with the clinical studies used for model validation. Participants received 500 mg clarithromycin in combination with 20 mg JP-1366 administered twice daily (b.i.d.) for five consecutive days. PK parameters including C_max_, T_max_ and AUC were collected after the last dose.

The PBPK model for clarithromycin was primarily adapted from Zhuan Yang et al. [23]. To improve alignment with reported clarithromycin-midazolam or triazolam DDI data, as well as the elimination characteristics of clarithromycin, K_I_ was optimized to 100 μM and P_app_ was optimized to 3.5 × 10^−6^ cm/s [24,25,26]. The optimized clarithromycin model has been internally validated. Predicted PK parameter ratios of JP-1366 were compared with observed data (ID: NCT05194046).

### 2.10. Prediction of Pharmacokinetic Changes in JP-1366 Caused by CYP Enzyme Modulators

The validated PBPK model was used to evaluate the potential risk of DDIs involving the CYP3A-mediated metabolic pathways. Various perpetrators were incorporated into the DDI model, encompassing strong CYP3A inhibitors (ketoconazole), moderate CYP3A inhibitors (fluconazole, fluvoxamine), a strong CYP3A inducer (rifampicin) and a moderate CYP3A inducer (fluvoxamine). Their PBPK models were pre-constructed in GastroPlus.

For the DDI prediction, both single and multiple doses of JP-1366 were co-administered with the various perpetrators. To evaluate the significance of DDIs, the adopted criteria were an area under the curve ratio (AUCR) < 0.5 (for induction) and >2 (for inhibition). A single 20 mg dose of JP-1366 was administered on Day 5 when co-administered with an inhibitor or on Day 8 with an inducer. Administration strategies of typical CYP enzyme modulators are listed in Table S3.

### 2.11. Prediction of the Inhibitory Effects of JP-1366 on CYP Substrates

The validated human PBPK model of JP-1366 was used to predict its effect as a perpetrator on typical CYP substrates, including omeprazole (CYP2C9, CYP2C19, CYP3A), warfarin (CYP2C9), rosiglitazone (CYP2C8, CYP2C9), atomoxetine (CYP2D6, CYP2C19), and midazolam (CYP3A). The corresponding PBPK models were pre-constructed in GastroPlus.

Briefly, healthy Chinese volunteers received 20 mg JP-1366 daily for 10 days, followed by a single dose of the victim drug (S-warfarin or midazolam or simvastatin or alfentanil). The in vitro inhibition parameters, including IC_50_ values against CYP enzymes, were obtained from studies conducted during the IND-enabling package and were incorporated into the PBPK model development. The corresponding parameters are summarized in Table S1. A single simulation trial with midazolam was performed under different f_u,mic_ values of JP-1366 (f_u,p_ and 1) to determine the impact of microsomal protein binding. PK parameters including C_max_ and AUC were collected after co-administration of both drugs. The DDI extent was determined by the AUC ratio in the co-administration group compared to that in the group with the victim drug only.

## 3. Results

### 3.1. Metabolite Profiles of JP-1366 and M1 in HLMs

**Figure 1:**
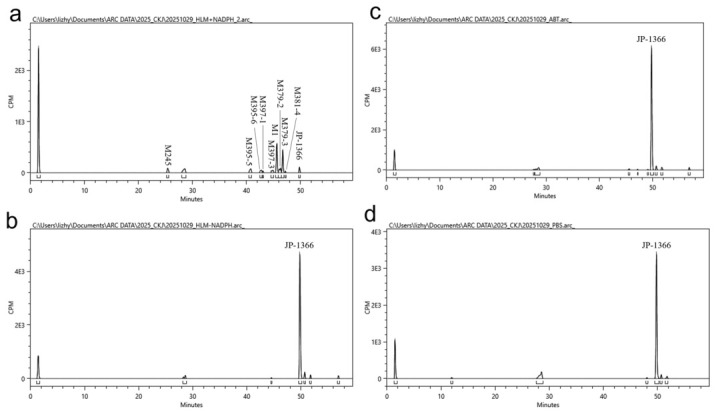
Radiochromatograms of [^14^C]JP-1366 and its metabolites in HLMs: (**a**) with NADPH; (**b**) without NADPH; (**c**) with NADPH and ABT; (**d**) PBS. “E3” in the figure denotes “×10^3^”.

A total of 17 phase I metabolites were detected in the NADPH-supplemented HLM incubation system using [^14^C]JP-1366 as the substrate. Based on the human metabolite identification data [18], these metabolites were identified as follows: the *N*-dealkylative metabolite M245; *N*-dealkylative and mono-oxidative metabolite M261-1; mono-oxidative and dehydrogenative metabolites M377-1 and M377-2; mono-oxidative metabolites M379-1 (M1), M379-2 and M379-3; mono-oxidative and hydrogenative metabolite M381-4; di-oxidative metabolites M395-1, M395-5, M395-6 and M395-7; di-oxidative and hydrogenative metabolites M397-1, M397-2 and M397-3; and tri-oxidative metabolites M411-4 and M411-6. Among them, M245, M1, M379-2, M379-3, M395-5 and M395-6 were identified as the major metabolites, accounting for 2.92%, 14.15%, 3.78%, 10.09%, 3.24% and 2.18% of the total radioactivity, respectively. M1, M379-3, M395-5 and M395-6 were also major metabolites identified in humans. The formation of these metabolites was completely inhibited in the presence of ABT, indicating that the phase I metabolism of JP-1366 was mainly mediated by P450 enzymes (Figure 1).

M411-3, one of the major metabolites identified in humans, was not detected in the HLM incubation system using JP-1366 as the substrate. To identify the enzymes responsible for the formation of M411-3 and elucidate the sequential relationships among the metabolites of JP-1366, in vitro metabolic incubations were conducted in HLMs supplemented with either NADPH or UDPGA, using M1 as substrate. A total of nine metabolites were detected, including *N*-dealkylative metabolite M245; *N*-dealkylative metabolite and mono-oxidative metabolites M261-1 and M261-2; di-oxidative metabolites M395-1, M395-4 and M395-5; di-oxidative and hydrogenative metabolite M397-1; tri-oxidative metabolite M411-3; and glucuronide conjugate M555-2. Among them, M245, M395-1, M395-5, M411-3 and M555-2 were the predominant components, and their formation exceeded that in the HLM incubation system using JP-1366 as the substrate.

### 3.2. Reaction Phenotyping of JP-1366

**Figure 2:**
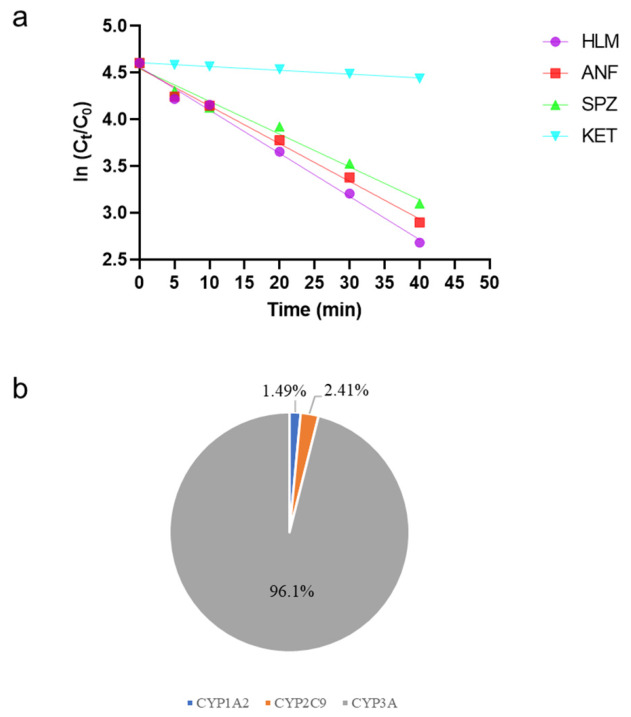
Contribution of individual CYP isoforms to JP-1366 metabolism in HLMs. (**a**) Semi-logarithmic plots of the natural logarithm of the remaining fraction of JP-1366 [ln(C_t_/C_0_)] versus incubation time (0–40 min) in the absence (HLM) or presence of selective CYP inhibitors: ANF (CYP1A2), SPZ (CYP2C9), AZA (CYP3A); (**b**) Corrected fractional contributions of CYP1A2, CYP2C9, and CYP3A to the metabolism of JP-1366, calculated using inhibitor-corrected matrix approach.

In the HLM incubation system with a protein concentration of 0.025–0.2 mg/mL, JP-1366 (1 µM) underwent linear depletion over 0–40 min. As shown in Figure 2a, the depletion of JP-1366 was markedly inhibited by azamulin, whereas α-naphthoflavone and sulfaphenazole showed only limited effects on JP-1366 depletion.

The potential off-target effects of the chemical inhibitors were evaluated using CYP marker reactions, and the results are summarized in Table 1. ANF inhibited the CYP1A2 marker reaction by 93.1%, while showing only minor inhibition of the CYP3A marker reaction. SPZ inhibited the CYP2C9 marker reaction by 94.3%, with 21.5% off-target inhibition of the CYP3A marker reaction. Azamulin inhibited the CYP3A marker reaction by 90.8%, with no apparent inhibition of CYP1A2 or CYP2C9 marker reactions.

Based on the JP-1366 substrate depletion rate constants, the uncorrected fractional contributions of CYP1A2, CYP2C9, and CYP3A were 12.8%, 24.0%, and 91.2%, respectively (Table 2). After coefficient matrix correction, the f_m_ of CYP1A2, CYP2C9, and CYP3A to JP-1366 metabolism were estimated to be 1.49%, 2.41%, and 96.1%, respectively (Figure 2 and Table 2).

### 3.3. Clinical DDI Between JP-1366 and Clarithromycin

The pharmacokinetic parameters of JP-1366 administered alone and in combination with clarithromycin are summarized in Table 3.

Under the T1 dosing regimen (20 mg JP-1366 capsule, bid for 5 days), the mean steady-state peak concentration (C_max,ss_) and area under the concentration–time curve over one dosing interval (AUC_τ_) were 165.42 ng/mL and 791.14 h·ng/mL, respectively. In contrast, under the T3 dosing regimen (20 mg JP-1366 capsule + 500 mg clarithromycin tablet + 1000 mg amoxicillin capsules, bid for 5 days), the corresponding values were 284.92 ng/mL and 1836.18 h·ng/mL. For the T1 regimen, the median time to reach peak concentration (T_max_) and mean elimination half-life (t_1/2_) were 1.00 h and 6.55 h, respectively, while those for the T3 regimen were 1.25 h and 11.78 h, respectively.

The geometric mean ratios (GMRs) with 90% confidence interval (90% CI) for JP-1366 (T3/T1) were 1.7766 (1.6397–1.9249) for C_max,ss_ and 2.4422 (2.2281–2.6768) for AUC_τ_. The 90% CIs for both C_max,ss_ and AUC_τ_ GMRs (T3/T1) fell outside the conventional bioequivalence range (0.8000–1.2500). Specifically, compared with the T1 regimen, the C_max,ss_ and AUC_τ_ under the T3 regimen increased by approximately 1.8-fold and 2.4-fold, respectively. When JP-1366 was co-administered with clarithromycin, the AUC_τ_ increased by more than 2-fold. Compared with JP-1366 administered alone, the GMR of t_1/2_ (T3/T1) was 1.71, less than 2-fold, indicating that clarithromycin affected the pharmacokinetics of JP-1366 through concurrent inhibition of intestinal and hepatic CYP3A-mediated metabolism.

### 3.4. PBPK Model Validation of JP-1366 Pharmacokinetics

**Figure 3:**
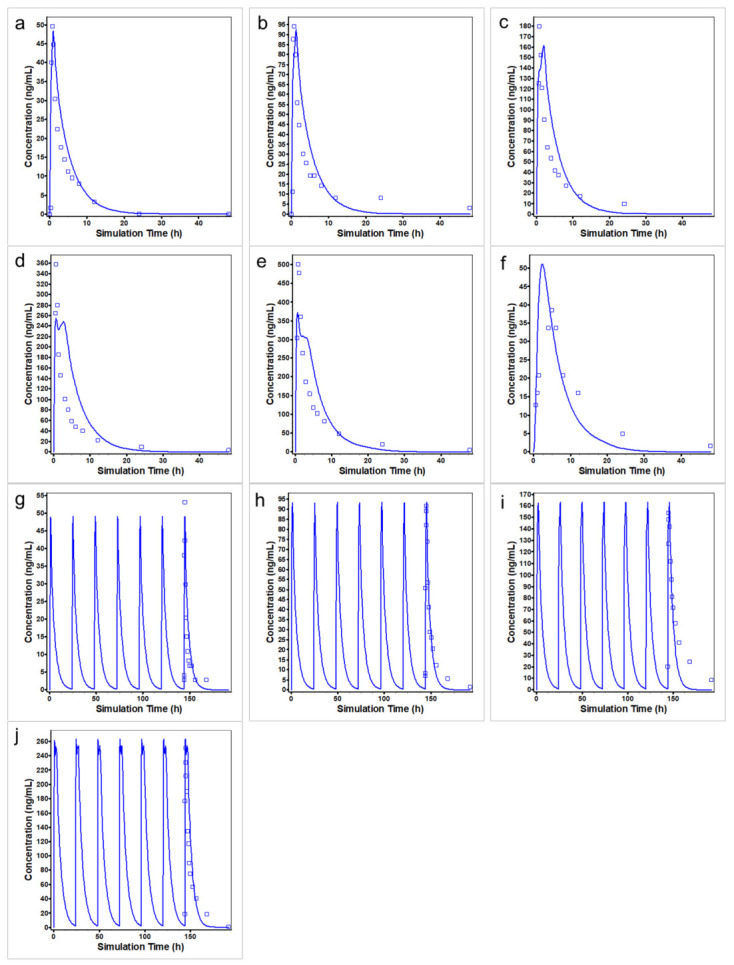
Simulated and observed plasma concentration–time profiles of JP-1366 in healthy subjects. (**a**–**e**): Healthy subjects with 5, 10, 20, 40, 60 mg single dose of JP-1366 under fasted condition; (**f**): Healthy subjects with 10 mg single dose of JP-1366 under fed condition; (**g**–**j**): Healthy subjects with 5, 10, 20, 40 mg once daily multiple doses of JP-1366 for 7 days under fasted condition. Blue squares represent the observed average plasma concentrations, and blue solid lines represent the PBPK model-simulated concentration–time profiles.

As presented in Figure 3, the model-simulated PK curves of JP-1366 following single or multiple doses were in good agreement with the observed concentrations. Fold errors (the ratio of predicted to observed values) were almost consistently within a two-fold range.

### 3.5. Simulation of JP-1366 Pharmacokinetics in JP-1366 DDI Study with Clarithromycin

The PBPK model predicted JP-1366 exposure ratios (when co-administered with 500 mg clarithromycin b.i.d. for five days) were in reasonable agreement with the observed results from the clinical study. The predicted ratios for C_max,ss_ and AUC_τ_ of JP-1366 deviated by no more than 20% from the observed values (prediction: AUC ratio = 2.81, C_max_ ratio = 1.77; observed: AUC ratio = 2.42, C_max_ ratio = 1.78). Overall, the simulation effectively recapitulated the observed effect of clarithromycin on JP-1366 exposure levels in the multiple-dose DDI study.

Additionally, the model predicted that both single and multiple doses of JP-1366 co-administered with clarithromycin demonstrated similar DDI ratios, although the multiple-dose DDI ratios were slightly higher than single-dose ones (multiple-dose vs. single-dose: AUC ratio = 2.81 vs. 2.75; C_max_ ratio = 1.77 vs. 1.66).

### 3.6. DDI Simulation of JP-1366 as a Victim Drug

The results of the DDI predictions are summarized in Table 4. The simulation demonstrated that ketoconazole, a strong CYP3A inhibitor, exerted the most pronounced DDI effect among all the simulated CYP modulators. Specifically, ketoconazole significantly altered the C_max_ and AUC of JP-1366: when JP-1366 was administered as a single dose, its C_max_ and AUC ratios (relative to JP-1366 alone) were found to be 2.20-fold and 3.80-fold, respectively. Comparable results were obtained with multiple doses of JP-1366, where the corresponding C_max_ and AUC ratios were 2.52-fold and 3.79-fold, respectively. In addition, co-administration of the strong CYP3A inducer, rifampicin, reduced the C_max_ and AUC of JP-1366 to 0.373-fold and 0.227-fold of the values with JP-1366 alone, respectively. The remaining simulated CYP3A modulators had no significant impact on JP-1366 exposure.

**Table 4:** Effects of CYP3A perpetrators on JP-1366 pharmacokinetics following single or multiple administration.

CYP3A Perpetrators		DDI Ratio			
		Single Dose		Multiple Dose	
		C_max_	AUC	C_max_	AUC
Strong inhibitor	Ketoconazole	2.20	3.80	2.52	3.79
Moderate inhibitor	Fluconazole	1.30	1.74	1.31	1.74
	Fluvoxamine	1.05	1.14	1.06	1.14
Strong inducer	Rifampicin	0.373	0.227	0.344	0.217
Moderate inducer	Efavirenz	0.647	0.501	0.623	0.493

### 3.7. DDI Simulation of JP-1366 as a CYP Perpetrator Drug

The DDI predictions between multiple doses of JP-1366 and a single dose of CYP substrates were performed, and the results are summarized in Table 5. The simulation showed that JP-1366 hardly altered the AUC and C_max_ of warfarin (a CYP2C9 substrate), omeprazole (a substrate of CYP2C9, CYP2C19, and CYP3A), rosiglitazone (a substrate of CYP2C8 and CYP2C9), or atomoxetine (a substrate of CYP2D6 and CYP2C19). In contrast, assuming consistent values for unbound fraction in microsomes (f_u,mic_) and unbound fraction in plasma (f_u,p_), JP-1366-mediated inhibition of CYP3A metabolism resulted in a significant interaction magnitude: the C_max_ and AUC ratios for midazolam (a CYP3A substrate) were 2.15-fold and 2.90-fold, respectively.

Sensitivity analyses were performed using simulations with different microsomal binding values (f_u,p_ and 1), revealing minor differences for most CYP substrates. An exception was midazolam, where notable differences were observed (f_u,mic_:f_u,p_ vs. 1:AUC ratio = 2.90 vs. 1.55; C_max_ ratio = 2.15 vs. 1.38).

## 4. Discussion

In this study, HLMs were utilized to clarify the reaction phenotyping of JP-1366 and evaluate its metabolic enzyme specificity. The major oxidative metabolites identified in humans, M379-1 (M1), M379-3, M395-5 and M395-6, were detected in the HLM incubation system with JP-1366 as the substrate, and these were also the primary metabolites in HLMs. Additionally, M411-3 and M555-2, major metabolites in humans, were successfully identified as the main metabolites in the HLMs incubated with M1 as the substrate. Collectively, the in vitro metabolic profile of HLMs showed a strong correlation with the in vivo metabolic profile in humans [18]. These results indicated that JP-1366 was primarily metabolized by P450 enzymes via initial mono-oxidation to form M1, followed by sequential reactions including further oxidation, glucuronidation, and *N*-dealkylation. Based on these data, mono-oxidation was estimated to account for more than 46.9% of the overall metabolic clearance of JP-1366.

Reaction phenotyping can be conducted using several approaches, among which chemical inhibition and recombinant enzyme systems are commonly used [27]. Although recombinant CYP enzyme systems provide high enzyme specificity, CYP expression levels, enzyme activities, and membrane environments in recombinant systems may differ from those in native human liver. Therefore, quantitative extrapolation from recombinant CYP data generally requires scaling factors such as RAF, ISEF, or CYP abundance, which may introduce additional uncertainty [28]. In contrast, chemical inhibition studies are usually conducted in HLMs or human hepatocytes, with more physiologically relevant enzyme composition. Therefore, the chemical inhibition approach was selected in this study to estimate CYP contributions.

Previous studies have reported that CYP1A2, CYP2C9, and CYP3A4 were mainly involved in the oxidative metabolism of JP-1366, particularly in the formation of M1 [17]. Given that JP-1366 exhibited high clearance in the HLM system and its metabolism therein showed good correlation with that in vivo, we employed the substrate depletion method combined with off-target effect correction to quantitatively evaluate the metabolic contributions of these three CYP isoforms. The results (Figure 2) showed that the contributions of CYP3A, CYP2C9, and CYP1A2 to the overall metabolic clearance of JP-1366 were 96.1%, 2.41%, and 1.49%, respectively. Notably, CYP3A emerged as the predominant enzyme mediating the metabolic clearance of JP-1366, consistent with other P-CAB class drugs.

However, it should be acknowledged that chemical inhibition-based reaction phenotyping may be affected by potential off-target inhibition. In addition, the f_m_ values estimated in this study were not further cross-validated using two orthogonal approaches. Further confirmation using recombinant CYP enzymes, human hepatocytes, or other complementary approaches would be helpful to strengthen the robustness of the quantitative assessment of individual CYP isoform contributions to JP-1366 metabolism.

Clinically available P-CABs, such as vonoprazan and tegoprazan, were commonly co-administered with amoxicillin and clarithromycin for *H. pylori* eradication therapy, in which clarithromycin acted as a strong CYP3A inhibitor [19,20]. Therefore, potential DDI involving JP-1366 during concomitant use warranted careful evaluation. Coadministration with clarithromycin increased the C_max_R and AUCR of 1.78 and 2.44 for JP-1366, respectively, indicating a moderate increase in systemic exposure, while the exposure remained within the observed safe exposure range. These findings are generally consistent with previously reported clinical DDIs for other P-CABs. For example, clarithromycin co-administration increased the C_max_R and AUCR of tegoprazan to approximately 1.65 and 2.50, respectively, whereas the corresponding values for vonoprazan were approximately 1.35 and 1.58, respectively [29,30]. Although P-CABs are generally considered CYP3A substrates with substantial CYP3A contribution, the magnitude of interaction observed with clarithromycin was lower than that typically reported for highly sensitive CYP3A substrates co-administered with strong CYP3A inhibitors. This observation may be explained, at least in part, by the tissue-specific inhibitory characteristics of clarithromycin as a mechanism-based, time-dependent CYP3A inhibitor. Previous studies have demonstrated that, under clinically relevant dosing regimens, clarithromycin generally produces a greater inhibitory effect on intestinal CYP3A4 activity than on hepatic CYP3A activity. This interpretation is also consistent with reported clinical DDI findings for clarithromycin and midazolam. Using a similar clarithromycin dosing regimen, the increase in midazolam exposure was substantially lower following intravenous administration than following oral administration, with AUC increases of approximately 2.7-fold and 7-fold, respectively, although midazolam is generally considered a sensitive CYP3A substrate with a CYP3A f_m_ close to 1 [31]. Collectively, these findings suggest that the observed increase in JP-1366 exposure is mechanistically consistent with the known inhibition characteristics of clarithromycin and the differential inhibition of intestinal versus hepatic CYP3A activity.

To expand the safety profile of clinical drug combinations, a PBPK model was established to predict DDIs with strong/moderate inhibitors of CYP3A. As with most PBPK models, several physiological and model-based assumptions were required. For example, gastrointestinal transit and regional intestinal conditions were described using model-based physiological parameters rather than subject-specific measurements, which may introduce some uncertainty in absolute exposure prediction. Nevertheless, the model-predicted Fa was consistent with human mass balance studies, supporting extensive absorption of JP-1366 and no apparent hydrolysis by gut microbiota after oral administration. The PBPK model described the in vivo clearance of JP-1366 by establishing kinetic equations for each metabolic enzyme, thereby describing its intestinal and hepatic metabolism, along with its renal excretion (which contributed minimally to total clearance). Due to the lack of effective and specific inhibitors for CYP3A4 or CYP3A5, coupled with the differential expression of CYP3A isoforms in the intestine and liver, it was not feasible to extrapolate intestinal CYP3A4 CL_int_ from hepatic CYP3A CL_int_ and hepatic-intestinal expression ratios [32,33]. Currently, hepatic first-pass metabolism in the model was based on hepatic clearance derived from IVIVE, while intestinal metabolism was optimized by fitting clinical PK data. Additionally, the literature indicates that intestinal first-pass metabolism of CYP3A substrates was also influenced by intestinal permeability [34]. In this study, the effective permeability coefficient (P_eff_) of JP-1366 was calculated via the Absca method using in vitro P_app_ values and validated with positive compounds from the same batch, which further supported the model’s ability to accurately characterize JP-1366 absorption [17]. Although the current model has been validated, incorporating intravenous PK data could further optimize the model and mitigate uncertainties arising from in vitro-in vivo discrepancies, considering the systematic relationship between hepatic clearance and absolute bioavailability.

Although the PBPK model developed in this study integrated available in vitro and clinical data to the extent possible, several limitations should be acknowledged in addition to the HLM-based extrapolation discussed above. First, the model utilized a surrogate population for simulation. Although model parameters were adjusted based on the demographic characteristics of clinical trial subjects, and this study primarily focused on the DDI evaluation of JP-1366, potential interethnic physiological differences may still exist [35]. Second, although the parameter optimization process was validated using multiple independent clinical datasets, uncertainties related to the model structure and parameterization cannot be completely ruled out [36]. Third, the current model was established and validated using data from healthy subjects; therefore, pharmacokinetic variability resulting from potential changes in physiological status and concomitant medication use in patient populations (particularly the elderly or patients with comorbidities) has not been fully evaluated [37,38,39]. Consequently, extrapolation to these populations remains subject to some uncertainty and requires further validation in future studies. Overall, since the model effectively describes the PK characteristics of healthy subjects, the mentioned limitations are expected to have a limited overall impact on the DDI prediction conclusions in this study. However, these limitations should still be taken into account when interpreting clinical extrapolation results and should be further evaluated using clinical outcome data when available.

Based on PBPK-DDI predictions, strong CYP3A inhibitors were predicted to markedly increase JP-1366 exposure, whereas moderate inhibitors were not expected to cause meaningful increases under the simulated conditions. Following 8 days of co-administration with rifampicin, JP-1366 plasma exposure was predicted to decrease significantly, with an AUC ratio of approximately 1:5, which may potentially reduce therapeutic efficacy. These results suggest that co-administration with strong CYP3A inhibitors may require close monitoring. Co-administration with moderate or weak CYP3A inhibitors was predicted to pose a relatively limited DDI risk, and co-administration with strong CYP3A inducers should be avoided or carefully evaluated.

We utilized the validated JP-1366 PBPK model to simulate DDIs when JP-1366 acts as a perpetrator. The reversible inhibitory IC_50_ values of JP-1366 were converted to fraction unbound of 1 or f_u,p_, respectively. Sensitivity analysis was then conducted to evaluate the impact of fu on DDI extent. Furthermore, although in-house in vitro data (unpublished) indicated JP-1366 had inductive and irreversible inhibitory effects on CYP3A, it also served as a CYP3A substrate and its PK parameters showed no significant changes after repeated dosing, suggesting induction and inactivation may counteract each other in vivo [40]. Therefore, in the absence of additional supporting evidence, this study only evaluated JP-1366’s reversible inhibition of CYP isoforms to avoid model instability and compromised extrapolative capability due to over-parameterization. PBPK predictions revealed comparable DDI risks for both conversion factor approaches. Even under the more conservative scenario using f_u,p_-adjusted unbound IC_50_ values, JP-1366 generally exhibited a low DDI risk as a perpetrator for most evaluated CYP pathways. Nevertheless, the predicted AUCR of approximately 2.9 for midazolam indicates a non-negligible interaction potential with sensitive CYP3A substrates. This result is consistent with the CYP3A-mediated interaction potential reported for another P-CAB, which increased midazolam exposure with an AUCR of 1.93, although the predicted effect of JP-1366 was numerically greater [30].

In summary, this study comprehensively elucidated the metabolic and excretory pathways of JP-1366 in humans. Additionally, through PBPK model-based simulations, it predicted the potential DDIs with CYP3A modulators, thereby providing scientific evidence to support its further development as a first-line therapy drug for GERD.

## Figures and Tables

**Table 1:** Off-target inhibition effect of chemical inhibitors on CYP marker reactions.

	Inhibitor	Selectivity of Inhibitors on CYP Marker Reactions (Percent Inhibition)		
		Phenacetin	Diclofenac	Testosterone
		CYP1A2	CYP2C9	CYP3A
CYP1A2	α-Naphthoflavone	93.1%	0.0%	11.3%
CYP2C9	Sulfaphenazole	5.56%	94.3%	21.5%
CYP3A	Azamulin	0.0%	0.0%	90.8%

Data are presented as the mean value of duplicate samples.

**Table 2:** Calculation of CYP fractional contributions to JP-1366 metabolism.

Incubation Group	*k* (min^−1^)	*f_m,uncorrected_* (%)	*f_m,corrected_* (%)
HLM (Control)	0.04622		
ANF (CYP1A2)	0.04031	12.8	1.49
SPZ (CYP2C9)	0.03511	24.0	2.41
AZA (CYP3A)	0.004065	91.2	96.1

**Table 3:** Effect of clarithromycin on the pharmacokinetics of JP-1366.

Parameter	Unit	T1	T3	GMR (T3/T1)	90% CI
C_max,ss_	ng/mL	165.42 (56.16)	284.92 (64.02)	1.7766	1.6397–1.9249
AUC_τ_	h·ng/mL	724.84 (302.64)	1836.18 (496.99)	2.4422	2.2281–2.6768
T_max_	h	1.00 (0.5–4.00)	1.25 (0.75, 3.00)		
t_1/2_	h	6.55 (2.39)	11.78 (6.14)		
CL_ss_/F	L/h	31.06 (18.52)	11.76 (3.68)		
Vd_ss_/F	L	268.29 (123.08)	184.66 (74.11)		

T1: 20 mg JP-1366 capsule, bid for 5 days. T3: 20 mg JP-1366 capsule + 500 mg clarithromycin tablet + 1000 mg amoxicillin capsules, bid for 5 days. Data are presented as arithmetic mean (SD) for C_max,ss_, AUC_τ_, t_1/2_, CL_ss_/F, and Vd_ss_/F, and median (min, max) for T_max_. GMRs and 90% confidence intervals were calculated based on log-transformed geometric mean values of C_max,ss_ and AUC_τ_.

**Table 5:** Effects of JP-1366 on the pharmacokinetics of CYP substrates in vivo.

		DDI Ratio			
		f_u,mic_ = 1		f_u,mic_ = f_u,p_	
Substrates	Metabolizing Enzyme	C_max_	AUC	C_max_	AUC
Omeprazole	CYP2C9, CYP2C19, CYP3A	1.019	1.027	1.069	1.104
Warfarin	CYP2C9	1.000	1.001	1.016	1.048
Rosiglitazone	CYP2C8, CYP2C9	1.000	1.002	1.033	1.098
Atomoxetine	CYP2D6, CYP2C19	1.000	1.001	1.038	1.055
Midazolam	CYP3A	1.378	1.554	2.146	2.896

f_u,mic_: fraction unbound in microsomes; f_u,p_: fraction unbound in plasma.

## Data Availability

The original contributions presented in this study are included in the article/Supplementary Materials. Further inquiries can be directed to the corresponding author.

## Supplementary Materials

The following supporting information can be downloaded at: https://www.mdpi.com/article/10.3390/pharmaceutics18060718/s1, Supplementary Method S1. LC-MS/MS conditions for CYP probe metabolites. Table S1. Final input parameters for JP-1366 PBPK model. Table S2. JP-1366 clinical study data used in PBPK model development and verification. Table S3. Summary of trial simulation design.

## Abbreviations

The following abbreviations are used in this manuscript:

ABT	1-Aminobenzotriazole
ANF	α-Naphthoflavone
AUC	Area under the concentration–time curve
AUC_τ_	Area under the concentration–time curve over a dosing interval
AUCR	Area under the curve ratio
b.i.d.	Twice a day
C_max_	Maximum plasma concentration
C_max,ss_	Maximum plasma concentration at steady state
CI	Confidence Interval
CYP	Cytochrome P450
DDI	Drug–drug interaction
f_m_	Fractional metabolic contribution
f_u,p_	Fraction unbound in plasma
f_u,mic_	Fraction unbound in microsomes
GMR	Geometric mean ratio
GERD	Gastroesophageal reflux disease
HLMs	Human liver microsomes
k	Depletion rate constant
KET	Ketoconazole
LC-MS/MS	Liquid chromatography–tandem mass spectrometry
NAB	Nocturnal acid breakthrough
NADPH	Nicotinamide adenine dinucleotide phosphate
P-CABs	Potassium-competitive acid blockers
P_app_	Apparent permeability
PBPK	Physiologically based pharmacokinetic
PBS	Phosphate-buffered saline
PK	Pharmacokinetics
PPIs	Proton pump inhibitors
SPZ	Sulfaphenazole
t_1/2_	Half-life
T_max_	Time to maximum concentration
UDPGA	Uridine 5′-diphosphoglucuronic acid
UPLC/Q-TOF MS	Ultra-performance liquid chromatography/quadrupole time-of-flight mass spectrometry
V_max_	Maximum velocity
V_ss_	Steady-state volume of distribution
