# Supplementary material for: CYP3A-Mediated Metabolism of Zastaprazan in Humans and Associated Drug–Drug Interactions
Source: Pharmaceutics. 2026 Jun 10;18(6):718. doi: 10.3390/pharmaceutics18060718 (PMC13305557; doi:10.3390/pharmaceutics18060718)
Supplement: Supplementary file 1 [file pharmaceutics-18-00718-s001.zip › pharmaceutics-4279740-supplementary.pdf]

Supplementary Method S1. LC-MS/MS conditions for CYP probe metabolites

Chromatographic separation was performed on a Kinetex C_{18} column (2.1 × 50 mm, 2.6 μm, 100 A). Mobile phase A consisted of 2 mM ammonium formate with 0.05% formic acid in water, and mobile phase B was methanol. The autosampler and column temperatures were maintained at 4 °C and 40 °C, respectively. Detection was performed using electrospray ionization in positive ion mode with multiple reaction monitoring.

For acetaminophen and 6β-hydroxytestosterone, gradient elution was performed at a flow rate of 0.520 mL/min as follows: 0.50 min, 12% B; 1.40–2.50 min, 95% B; 2.60 min, 12% B; and 3.50 min, stop. The injection volume was 3.00 μL. For 4′-hydroxydiclofenac, gradient elution was performed at a flow rate of 0.550 mL/min as follows: 0.70 min, 12% B; 1.80–2.05 min, 85% B; 2.10–2.50 min, 95% B; 2.60 min, 12% B; and 3.50 min, stop. The ion source parameters were as follows: curtain gas, 9 psi; ion source gas 1, 40 psi; ion source gas 2, 40 psi; temperature, 500 °C; ion spray voltage, 5500 V.

The MRM transitions were m/z 152.1 → 110.0 for acetaminophen with CE being 26 V, m/z 312.1 → 231.0 for 4′-hydroxydiclofenac with CE being 45 V, and m/z 305.2 → 269.2 for 6β-hydroxytestosterone with CE being 33 V.

For acetaminophen, the calibration range was 1.00–1000 ng/mL, with QC concentrations of 3.00, 30.0, 400, and 800 ng/mL. For 6β-hydroxytestosterone, the calibration range was 5.00–5000 ng/mL, with QC concentrations of 15.0, 150, 2000, and 4000 ng/mL. For 4′-hydroxydiclofenac, the calibration range was 10.0–2000 ng/mL, with QC concentrations of 30.0, 150, 800, and 1600 ng/mL.

Each analytical run included calibration standards and QC samples at four concentration levels. The predefined run acceptance criteria were as follows: the back-calculated concentrations of calibration standards should be within ±15% of the nominal concentrations, except for the lower limit of quantification (LLOQ), for which ±20% was accepted. For QC samples, the accuracy should be within ±15% of the nominal concentrations and the precision should not exceed 15%. Analytical runs not meeting these criteria were not accepted for data interpretation. All runs used for the assessment of CYP inhibitor selectivity and inhibitory efficiency met the predefined acceptance criteria.

Supplementary Table S1. Final input parameters for JP-1366 PBPK model

	Parameter	Description	Unit	Value	Source
PBPK	MW	molecular weight		362.48	a
	$f_{u,p}$	fraction of unbound drug in plasma	%	1.13	b
	Log P	water partition coefficient		1.72	b
	pka	dissociation constant		5.81 (Base)	b
	Solubility	Reference solubility	mg/mL	0.002	b
	Solubility @ pH	pH of Reference solubility		6.8	
	Precipitation Time		s	900	c
	B/P ratio	blood-to-plasma ratio		0.55	b
	Kp calculation method	tissue-to-plasma drug partition coefficient method		Poluin & Theil - Homogeneous	d
	P_{app}	apparent permeability coefficient	e-5 cm/s	2.3	e
	P_{eff}	effective permeability	e-4 cm/s	3.55	f
	CL - hepatic				
	CYP3A-k_m	Michaelis constant	µM	13.7	g
	CYP3A-V_{max}	Maximum velocity of enzyme reaction	nmol/min/mg protein	7.91	h
	CYP2C9-k_m	Michaelis constant	µM	4.3	g
	CYP2C9-V_{max}	Maximum velocity of enzyme reaction	pmol/min/mg protein	58.8	h
	CYP1A2-k_m	Michaelis constant	µM	21	g

	CYP1A2-V_{max}	Maximum velocity of enzyme reaction	pmol/min/mg protein	183	h
	CL - intestine				
	CYP3A4-k_m	Michaelis constant	μM	13.7	g
	CYP3A4-V_{max}	Maximum velocity of enzyme reaction	pmol/min/mg protein	219	i
	CYP2C9-k_m	Michaelis constant	μM	4.3	g
	CYP2C9-V_{max}	Maximum velocity of enzyme reaction	pmol/min/mg protein	147.5	h
	CL-kidney				
	CL_r	renal CL	L/h	0.002	j
DDI	CYP3A	IC_{50}	μM	4.14	b
	CYP2C8	IC_{50}	μM	14.4	b
	CYP2C9	IC_{50}	μM	1.32	b
	CYP2C19	IC_{50}	μM	22.3	b
	CYP2D6	IC_{50}	μM	20.8	b

a: Data from Drug Bank. b: From studies conducted during the IND-enabling package. c: Default value of GastroPlus. d: Built in method of GastroPlus, which matched clinical observation PK data. e: From literature [17]. f: Transported data using built in method of Absca, which was validated using positive analyte, g: From literature, average [17]. h: Calculated data were based on "$V_{max} = CL_{int} / k_m$", CL_{int} was calculated based on fractional metabolic contribution f_m and the in vitro hepatic microsomal metabolic stability of 600 μL/min/mg in house data. i: Estimated data from clinical PK data. j: calculated data from clinical PK data based on "$CL_r = Ae_{urine} / AUC$".

Supplementary Table S2. JP-1366 clinical study data used in PBPK model development and verification

Clinical study	Population	Study description	Dose regimen	PK data used in PBPK model
NCT03383042	Healthy volunteer	SAD of Phase 1 dose escalation	5 mg (SD)	Verification
	Mean age of 27.5 years		10 mg (SD)	Verification
	Mean weight of 72.5 kg		10 mg, fed (SD)	Verification
	Mean BMI of 23.7 kg/m^2		20 mg (SD)	Development
			40 mg (SD)	Verification
			60 mg (SD)	Verification
	Healthy volunteer	MAD of Phase 1 dose escalation	5 mg (qd)	Verification
	Mean age of 28.8 years		10 mg (qd)	Verification
	Mean weight of 70.0 kg		20 mg (qd)	Verification
	Mean BMI of 23.1 kg/m^2		40 mg (qd)	Verification
NCT05194046	Healthy volunteer	Clinical DDI study with strong CYP3A inhibitor	20 mg bid (DDI with clarithromycin)	Verification

SD: Single dose. Qd: Multiple doses, one daily. SAD, Single ascending dose. MAD, Multiple ascending dose.

PK data were detected on Day 7.

Supplementary Table S3. Summary of trial simulation design

Precipitant	Object	Dosing Regimen
Inhibitor (clarithromycin)	JP-1366 (MD)	clarithromycin 500 mg bid
Inhibitor (clarithromycin)	JP-1366 (SD)	clarithromycin 500 mg bid
Inhibitor (ketoconazole)	JP-1366 (SD)	ketoconazole 400 mg qd
Inhibitor (fluconazole)	JP-1366 (SD)	fluconazole 200 mg qd
Inhibitor (fluvoxamine)	JP-1366 (SD)	fluvoxamine 50mg qd
Inducer (rifampicin)	JP-1366 (SD)	rifampicin 600 mg qd
Inducer (efavirenz)	JP-1366 (SD)	efavirenz 400 mg qd
